# Transcriptional adaptation of *Mycobacterium ulcerans* in an original mouse model: New insights into the regulation of mycolactone

**DOI:** 10.1080/21505594.2021.1929749

**Published:** 2021-06-09

**Authors:** Marie Robbe-Saule, Mélanie Foulon, Isabelle Poncin, Lucille Esnault, Hugo Varet, Rachel Legendre, Alban Besnard, Anna E. Grzegorzewicz, Mary Jackson, Stéphane Canaan, Laurent Marsollier, Estelle Marion

**Affiliations:** a Univ Angers, Inserm, CRCINA, Angers, France; b Aix-Marseille Université, CNRS, LISM, Marseille, France; c Plate-forme Transcriptome Et Epigenome, Biomics, Centre De Ressources Et Recherches Technologiques (C2RT), Institut Pasteur, Paris, France; d Hub De Bioinformatique Et Biostatistique - Département Biologie Computationnelle, Institut Pasteur, Paris, France; e Mycobacteria Research Laboratories, Department of Microbiology, Immunology and Pathology, Colorado State University, Fort Collins, Colorado, United States

**Keywords:** *Mycobacterium ulcerans*, host-bacterium interaction, mycolactone, rna-sequencing, metabolism

## Abstract

*Mycobacterium ulcerans* is the causal agent of Buruli ulcer, a chronic infectious disease and the third most common mycobacterial disease worldwide. Without early treatment, *M. ulcerans* provokes massive skin ulcers, caused by the mycolactone toxin, its main virulence factor. However, spontaneous healing may occur in Buruli ulcer patients several months or years after the disease onset. We have shown, in an original mouse model, that bacterial load remains high and viable in spontaneously healed tissues, with a switch of *M. ulcerans* to low levels of mycolactone production, adapting its strategy to survive in such a hostile environment. This original model offers the possibility to investigate the regulation of mycolactone production, by using an RNA-seq strategy to study bacterial adaptation during mouse infection. Pathway analysis and characterization of the tissue environment showed that the bacillus adapted to its new environment by modifying its metabolic activity and switching nutrient sources. Thus, *M. ulcerans* ensures its survival in healing tissues by reducing its secondary metabolism, leading to an inhibition of mycolactone synthesis. These findings shed new light on mycolactone regulation and pave the way for new therapeutic strategies.

## Introduction

*Mycobacterium ulcerans* (*M. ulcerans*) is the causal agent of Buruli ulcer, a chronic infectious disease and the third most common mycobacterial disease after tuberculosis and leprosy [1]. This disease is characterized by skin lesions, caused by the major virulence factor of *M. ulcerans*, the toxin mycolactone. *M. ulcerans* infection has three stages: (i) a pre-ulcerative stage, (ii) an ulcerative stage, during which lesions progress toward massive ulcerations, and (iii) spontaneous healing after long periods of ulceration, leading to severe sequelae, in some patients [1,2]. About 25% of infected subjects, especially children, become permanently disabled and endure permanent developmental, economic, and social difficulties [3–5].

The toxin mycolactone is a very original lipid toxin with pleiotropic effects. Its cytotoxicity provokes the cutaneous lesions that are the main clinical features of Buruli ulcer [6,7]. However, mycolactone has other cellular activities, facilitating host colonization by this bacillus [8]. At non-cytotoxic concentrations, mycolactone also modulates the immune system, modifying cytokine production and acting on the peripheral nervous system to induce the formation of painless lesions [9–11]. This toxin is a distinctive feature of *M. ulcerans* and plays a key role in its eco-epidemiology and pathogenesis.

Relevant and robust preclinical models of bacterial infection are powerful tools for deciphering pathogenesis and identifying new therapeutic strategies [12–15]. We previously developed a murine model of *M. ulcerans* infection based on the use of inbred FVB/N mice, which display each stage of infection, from early lesions to spontaneous healing, for studies of the pathways underlying bacterial persistence [16,17]. With this original mouse model, we showed that the bacteria remained present, in a viable state and at high loads in healed tissues, but switched to a low mycolactone production phenotype, suggesting the adoption of an alternative strategy for survival in this hostile environment.

We now need to dissect the mechanisms of regulation for mycolactone synthesis, to pave the way for the development of new therapeutic strategies. Here, we used a whole-genome microarray analysis for *Mycobacterium ulcerans* from *in vivo* sites of infection, together with electron microscopy and *in vitro* analyses, to decipher the metabolic changes involved in mycolactone regulation.

## Materials and methods

### Ethics statements for animal experiments

All animal experiments were performed in full compliance with national (articles R214-87 to R214-90 from the French “rural code”) and European (directive 2010/63/EU of the European Parliament and of the Council of 22 September 2010 on the protection of animals used for scientific purposes) guidelines. All protocols were approved by the ethics committee of region Pays de la Loire under protocol nos. CEEA 2009.14 and 2012.145. Mice were maintained under specific pathogen-free conditions in the animal house facility of Angers University Hospital, France (agreement A 49 007 002).

### M. ulcerans *strain and inoculation*

*Mycobacterium ulcerans* strain 01G897 was originally isolated from patients from French Guiana [18]. A bacterial suspension was prepared as previously described [16,19], and its concentration was adjusted to 2 × 10^5^ acid-fast bacilli/mL for the inoculation of 1 × 10^4^ bacilli in a volume of 50 µL into the tails of six-week-old female inbred FVB/N mice (Charles River Laboratories, Saint-Germain-Nuelles, France).

### In vitro *growth conditions of* M. ulcerans *in MGIT*

*M. ulcerans* strain 01G897 was also used to study growth and mycolactone production in different carbon sources. Tubes containing BBL MGIT medium (BD BACTEC) supplemented with 10% OAC (oleic acid, albumin, catalase)/MGIT PANTA antibiotic mixture, were supplemented with 0.2% glucose or 0.2% acetate, or 0.2% lactate. Media were inoculated with 10^2^ bacteria and incubated at 30°C for three months.

### Mycolactone quantification

The samples were centrifuged at 3,200 x *g* for 30 min and pellets were resuspended in 5 mL of a chloroform/methanol mixture (2:1, v/v) and incubated overnight at 4°C in dark conditions. Cell debris was removed by centrifugation and the Folch reaction was initiated by adding 0.2 volumes of water. The organic phase was dried and resuspended in 200 µL ethanol. Mycolactone quantity was determined in the acetone-soluble total lipid fraction. Its concentration was determined by measuring absorbance (λ_max_ = 362 nm, logε = 4.29), and purity was evaluated by high-performance liquid chromatography (HPLC). The mycolactone peak was quantified by calculating the area under the curve and is expressed relative to the number of bacteria (CFU) in the corresponding sample.

### Bacterial load determination

*M. ulcerans* load was evaluated by counting CFU. Samples were homogenized lightly with a TissueRuptor to disrupt aggregates, and serial dilutions of 10^−1^ to 10^−8^ were prepared. We used 200 µL of each dilution to inoculate Löwenstein–Jensen slants (BD Biosciences), and CFU were counted after eight weeks of incubation at 30°C.

### RNA extraction and purification

***In vivo* experiments**. RNA was extracted from infected tail skin at the edema stage (30 days post-infection) and at the healing stage (60–75 days post-infection), by the differential lysis method, as previously described [17]. Briefly, tail skin from infected mice was excised and broken up with a TissueRuptor (Qiagen). Tissue homogenates were digested with a proteinase K solution (Qiagen) for 10 min at 55°C and centrifuged at 3,200 x g for 15 min. The pellets, which contained the bacterial cells, were resuspended in 300 µL TRI reagent (Zymo Research) and 300 µL RLT buffer (Qiagen) supplemented with 1% β-mercaptoethanol. The samples were transferred to a bead beating tube (0.1 mm glass beads, MoBio) and shaken with TissueLyser (Qiagen) for 5 min at 30 Hz. The samples were centrifuged at 10,000 x g for 5 min to remove cell debris, and the supernatant was transferred to a clean tube containing 1 volume of 100% ethanol. RNA was purified and treated with DNase, with the Direct-zol RNA MiniPrep Kit (Zymo Research), according to the manufacturer’s protocol, and was eluted in 50 µL RNase- and DNase-free water. RNA quality and integrity were assessed with the Experion automated electrophoresis system (Bio-Rad) (Additional file 1).

***In vitro* cultures**. The samples were centrifuged at 3,200 x *g* for 20 min and pellets were resuspended in 500 µL TRI reagent (Zymo Research). The samples were transferred to a bead beating tube (0.1 mm glass beads, MoBio) and shaken with TissueLyser (Qiagen) for 5 min at 30 Hz. The samples were centrifuged at 10,000 x *g* for 5 min to remove cell debris, and the supernatant was transferred to a clean tube containing 1 volume of 100% ethanol. RNA was purified and treated with DNase, with the Direct-zol RNA MiniPrep Kit (Zymo Research), according to the manufacturer’s protocol, and was eluted in 30 µL RNase- and DNase-free water.

### Transcriptional analysis (RNA-seq/RT-qPCR)

#### In vivo *experiments: RNA sequencing*

**(i) Library preparation and sequencing**: Ribosomal RNA was first removed from the total RNA with the RiboZero Epidemiology Illumina kit, and libraries were prepared with the TruSeq Stranded Total RNA LT Sample Prep kit (Illumina), according to manufacturer’s protocol. Library quality was checked with the DNA-1000 kit (Agilent) on a 2100 Bioanalyzer, and quantification was performed with Quant-It assays on a Qubit 1.0 fluorometer (Invitrogen). Clusters were generated for the resulting libraries, with Illumina HiSeq SR Cluster Kit v4 reagents. Sequencing was performed with the Illumina HiSeq 2500 system and HiSeq SBS kit v4 reagents. Runs were performed over 65 cycles, including seven indexing cycles, to obtain 65 bp single-end reads. **(ii) Bioinformatics analysis**: Reads were cleaned to remove adapter sequences and low-quality sequences with an in-house program (https://github.com/baj12/clean_ngs). Only sequences at least 25 nt in length were considered for further analysis. Bowtie version 1.2.2 [20] was used, with default parameters, to generate an alignment with the reference genome (*Mycobacterium ulcerans* Agy99, CP000325.1, from NCBI). Genes were counted with feature Counts version 1.4.6-p3 [21] from the Subreads package (parameters: -t gene – g ID – s 1). Count data were analyzed with R version 3.3.1 [22] and the Bioconductor package DESeq2 version 1.12.3 [23]. Normalization and the estimation of dispersion were performed with DESeq2, using the default parameters, but statistical tests for differential expression were performed without applying the independent filtering algorithm. A generalized linear model was used to test for differential expression between biological conditions (healed versus edema stage). For each pairwise comparison, raw *p*-values were adjusted for multiple testing according to the Benjamini and Hochberg (BH) procedure [24] and genes with an adjusted *p*-value lower than 0.05 were considered to be differentially expressed. For pathway enrichment analysis, we used the Biocyc tool (https://biocyc.org/) with a *p*-value <0.05 in Fisher’s exact test. **(iii) Data accessibility**: The data discussed in this publication have been deposited in NCBI’s Gene Expression Omnibus and are accessible through GEO Series accession number GSE157956 (https://www.ncbi.nlm.nih.gov/geo/query/acc.cgi?acc=GSE157956).

#### In vitro *cultures: RT-qPCR*

**(i) Reverse transcription (RT**): The first-strand cDNA was synthesized in a reaction volume of 20 µL containing 100 ng total RNA, 500 ng random primers (Invitrogen) and the M-MLV reverse transcriptase (Invitrogen). We checked for the presence of contaminating DNA in RNA samples, by performing a negative control with no reverse transcriptase (RT-) for each sample. **(ii) Quantitative real-time PCR (qPCR**): qPCR was performed in a reaction volume of 10 µL containing Maxima SYBR Green qPCR Master mix (Thermo Scientific), 300 nM primers and 2.5 µL of a two-fold dilution of cDNA/RT-. The sequences of the primers used are provided in Table S1. Reactions were run on a AriaMx Thermocycler (Agilent), with the following program: 10 min at 95°C followed by 40 cycles of 15 s at 95°C and 30 s at 60°C. Relative fold-changes in gene expression were assessed by the ∆∆Ct method, with the *fadD28* gene as the housekeeping gene and normalization against *M. ulcerans* cultured in the presence of glucose. Statistical analysis was performed with GraphPad Prism 7 (version 7.02, GraphPad Software, San Diego, CA, USA). Differences between two groups were assessed in nonparametric Mann–Whitney *U* tests. Significant differences are illustrated as *p < 0.05, **p < 0.01, ***p < 0.001.

## Transmission electron microscopy

***In vivo* experiments**. Immediately after the mice were killed (at the edema or healing stage), skin from around the lesions was carefully excised and cut into small pieces. Samples were fixed by overnight incubation at room temperature with 2.5% glutaraldehyde (Sigma) in 0.1 M sodium cacodylate buffer (pH 7.2) containing 0.1 M sucrose, 5 mM CaCl_2_ and 5 mM MgCl_2_ (complete cacodylate buffer). Tissues were washed in complete cacodylate buffer, and post-fixed by incubation for 1 h with 1% osmium tetroxide in the same buffer. They were then washed three times in complete cacodylate buffer, dehydrated in a graded series of ethanol solutions and gradually incorporated into Spurr resin. Thin sections (80 nm thick) were stained with 1% uranyl acetate in distilled water, and then with lead citrate, for observation by electron microscopy. Image acquisition was performed with a FEI Tecnai G2 20 TWIN 200 kV transmission electron microscope, with a Lab6 cathode and an Eagle 2k camera. Images were processed with the open-source program ImageJ 1.51 K (NIH, USA). We examined between 67 and 118 mycobacteria per stage (edema versus healing stage) to determine the percentage of *M. ulcerans* profiles of different categories.

***In vitro* cultures**. The samples were centrifuged at 3,200 x *g* for 20 min and the pellets were resuspended with PBS-Tween20 (0.05%) and washed with PBS. Bacteria were fixed 30 min with 5% glutaraldehyde and 2 h with 2.5% glutaraldehyde in 0.1 M sodium cacodylate buffer (pH 7.2) containing 0.1 M sucrose, 5 mM CaCl_2_ and 5 mM MgCl_2_ (complete cacodylate buffer), at room temperature. Bacteria were washed in complete cacodylate buffer, and post-fixed by incubation overnight with 1% osmium tetroxide in the same buffer. They were then washed three times in complete cacodylate buffer, dehydrated in a graded series of ethanol solutions and gradually incorporated into Spurr resin. Thin sections (80 nm thick) were stained with 1% uranyl acetate in distilled water, and then with lead citrate, for observation by electron microscopy. Image acquisition was performed with a FEI Tecnai G2 20 TWIN 200 kV transmission electron microscope, with a Lab6 cathode and an Eagle 2k camera.

## Glucose and lactate quantification

We excised 100 mg of skin from around the lesions immediately after the mice were killed. The skin was cut into small pieces in a Petri dish containing 500 µL PBS supplemented with cOmplete, EDTA-free protease inhibitor cocktail (Roche). Tissue samples were transferred to a 15 mL conical tube, and the Petri dish was washed with 500 µL PBS supplemented with protease inhibitor cocktail. The tissues were crushed with a TissueRuptor (Qiagen) for 1 min on ice. The resulting suspensions were centrifuged at 10,000 x *g* for 5 min at 4°C and the supernatant was carefully recovered in a 1.5 mL microtube. Endogenous enzymatic activity was prevented during the determination, according to the manufacturer’s protocol, by subjecting samples to deproteinization, by adding 250 µL 4 M trichloroacetic acid (final molarity of 1 M). The tubes were briefly vortexed and incubated on ice for 5 min. Precipitated proteins were eliminated by a centrifugation for 2 min, at 12,500 x *g* and 4°C. We added 2 M potassium hydroxide to the supernatant, in an amount equivalent to 34% of the total volume, to neutralize the trichloroacetic acid. Samples were briefly vortexed and centrifuged for 15 min, at 12,500 x *g* and 4°C. The final deproteinized supernatant was recovered and stored at −20°C until use for determinations. Glucose and lactate were quantified with a glucose assay kit (ab65333) and an L-lactate assay kit (ab65331) from Abcam, according to the manufacturer’s protocol.

## Results

By using our original mouse model of spontaneous healing, we study the switch to a low mycolactone production phenotype in *M. ulcerans* [16]. After developing and optimizing method for extracting bacterial RNA from mouse skin tissues colonized with *M. ulcerans* [17], we used an RNA-seq strategy to determine the transcriptional signatures of *M. ulcerans* during acute infection and at the healing stage.

## Genes differentially expressed in *M. ulcerans* during *in vivo* infection

**Functional categorization of genes**

**Figure 1.**
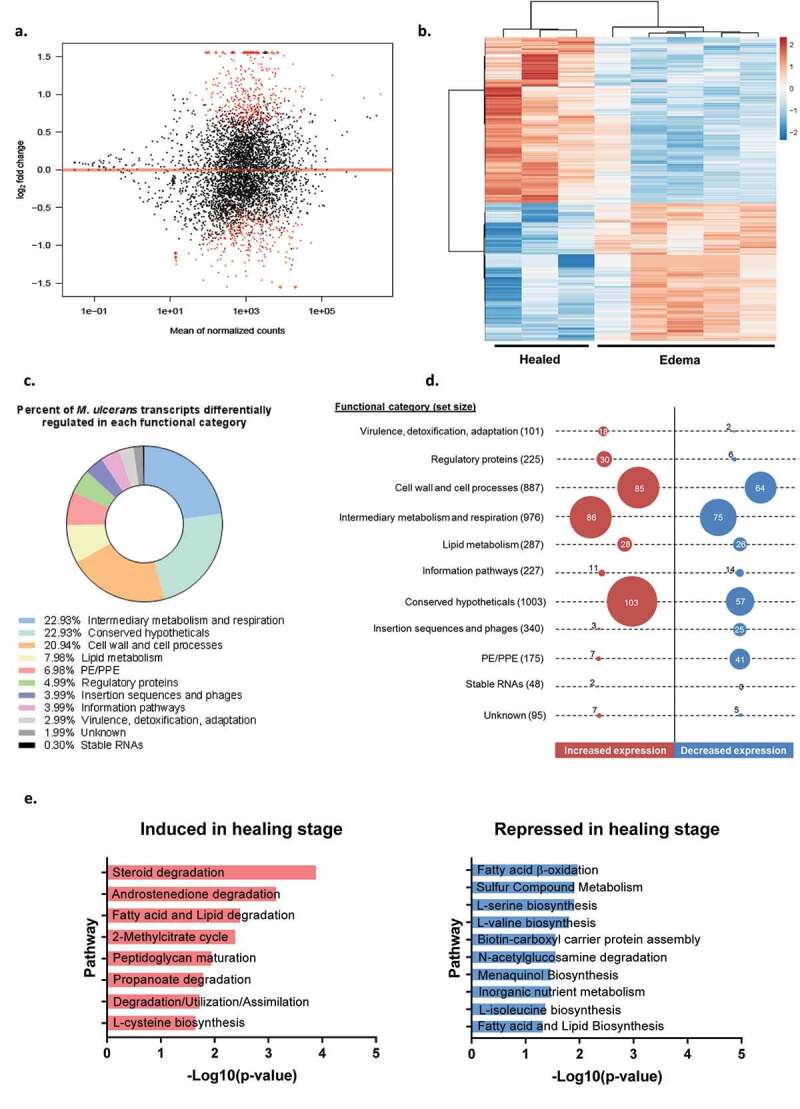
**RNA-seq analysis of *M. ulcerans* in the FVB/N mouse spontaneous healing model**. (a) MA-plot of the data for the comparison of the healing (*n = *3 mouse) and edema (*n = *5 mouse) stages. Differentially expressed genes, identified with a false discovery rate of < 0.05, are highlighted in red. (b) Heatmap of the 707 differentially expressed genes (DEGs) of *M. ulcerans* isolated at the healing and edema stages in a mouse model. The heatmap is based on the variance-stabilized transformed count matrix, the rows and columns of which were re-ordered by hierarchical clustering (using the correlation and Euclidean distances, respectively, and the Ward aggregation criterion). The color scale ranges from −2 to +2 as the rows of the matrix have been centered. (c) The pie chart shows the percentage of differentially expressed genes in each functional category as per the BuruList database. (d) The numbers of differentially expressed genes are shown by functional category, according to BuruList database. Circle sizes are proportional to the number of genes with significant differential expression (adjusted *p*-value < 0.05) between the edema and healing stages. The total number of genes per functional category listed in BuruList is indicated in parentheses. (e) Pathway enrichment analysis for genes that are induced (left panel) or repressed (right panel) during the healing stage. Negative log_10_(*p*-values) were calculated from the difference between the observed and expected numbers of induced or repressed genes annotated in the Biocyc database. Only significant pathways (Fisher’s exact test) with *p-*value < 0.05 are represented

We performed a comparative transcriptomic analysis of *M. ulcerans* on purified RNA from mouse skin tissues at the edema and healing stages. In total, 707 differentially expressed genes (DEGs) were identified, after filtering on a false discovery rate (FDR) < 0.05 (Additional file 2). These DEGs included 387 genes that were upregulated and 320 that were downregulated at the healing stage relative to the edema stage (Figure 1a and 1b). We sorted these DEGs on the basis of functional category designation, using the Burulist database (Figure 1c and 1d). Most of the differentially expressed genes belonged to the categories of intermediary metabolism and respiration (23%, 86 upregulated, 75 downregulated), conserved hypothetical proteins (23%, 103 upregulated, 57 downregulated) and cell wall and cell processes (21%, 85 upregulated, 64 downregulated) (Figure 1c and 1d). About 4–8% of the differentially expressed genes belonged to the categories of lipid metabolism (28 upregulated, 26 downregulated), PE/PPE families (7 upregulated, 41 downregulated), regulatory proteins (30 upregulated, 6 downregulated), insertion sequences and phages (3 upregulated, 25 downregulated), or information pathways (11 upregulated, 14 downregulated) (Figure 1c and 1d). The remaining 0.3–3% belonged to the virulence, detoxification, adaptation (18 upregulated, 2 downregulated) or RNA (2 upregulated) functional categories (Figure 1c and 1d). We then performed a pathway enrichment analysis of DEGs with the BioCyc database.

**Pathway enrichment analysis**

This analysis identifies pathways likely to be significantly induced or repressed (*p*-value < 0.05) (Figure 1e). The *M. ulcerans* genome remains poorly annotated. We therefore took advantage of the close phylogenetic relationship between *M. tuberculosis* and *M. ulcerans* [25] to perform this analysis. Indeed, using the KEGG Orthology database, we found *M. tuberculosis* orthologs for each significantly DEG of *M. ulcerans*. From the 707 DEGs, we selected 374 genes from *M. tuberculosis* with a percentage identity >65%, as a means of identifying enriched pathways (Additional file 3).

Lipid metabolism was found to be the major pathway induced during the healing stage. Upregulation was observed for steroid, fatty acid and lipid degradation, but also for methylcitrate cycle (MCC) and propanoate degradation (Figure 1e). Indeed, the breakdown products of fatty acids (acetyl-CoA and propionyl-CoA) are probably metabolized via the citric acid and methylcitrate cycle (MCC), as suggested by the strong induction of the *gltA1* and *mul*_*2496*, genes, with fold-changes (FC) in expression of 7.0 (FDR = 1.26E-11) and 6.8 (FDR = 5.45E-25), respectively. The proteins encoded by *gltA1* and *mul_2496* are similar to those encoded by *prpC* and *prpD* in *M. tuberculosis*, which participate in the catabolism of propionyl-CoA. The RNA-seq data also revealed induction of the *gloA_1* (*rv1322a*) gene, encoding a protein involved in the methylmalonyl-CoA pathway (MMC), with a FC of 2.0 (FDR = 0.008). These two pathways, MCC and MMC, are usually associated with propionate metabolism/fatty acid metabolism.

On the other hand, some of the pathways repressed during healing stage also related to lipid metabolism (Figure 1e). More interestingly, we observed a downregulation of expression for a gene encoding an acetyl-CoA carboxyl transferase, *accD3* (FC = 0.57, FDR = 0.011), and a gene encoding a biotin carboxylase, *accA1* (FC = 0.59, FDR = 0.036). The product of *accD3* is specifically involved in the biosynthesis of malonyl-CoA, an elementary substrate for lipid biosynthesis. Thus, the induction of the methylcitrate cycle and the methylmalonyl-CoA pathway, and the repression of genes involved in the malonyl-CoA pathway, suggest that fatty-acid metabolism plays a key role in the adaptation of *M. ulcerans* in healed tissues.

Furthermore, the repressed pathways included, in particular, pathways involved in the degradation of sugar derivatives and the biosynthesis of amino acids, fatty acids and lipids (Figure 1e), suggest an important nutrient deficiency. This hypothesis is supported by the induction of *mul_0949* (FC = 5.6, FDR = 1.16E-19), encoding a potential sugar transporter (*rv1200*), and *lipU* (FC = 1.7, FDR = 0.043), encoding a putative lipase, both of which have been reported to be upregulated under nutrient stress [26,27]. Further evidence for a stressful environment is provided by the induction of (i) *relA* (FC = 1.6, FDR = 0.010), encoding a putative regulator of the stringent response induced under starvation, (ii) *mprA* (FC = 1.9, FDR = 0.009), *phoP* (FC = 1.7, FDR = 0.039), *regX3* (FC = 2.6, FDR = 1.18E-5) expressing elements of two-component systems, and a sigma factor, *sigB* (FC = 1.7, FDR = 0.009), all well-characterized regulators of *M. tuberculosis* responsible for favoring survival under various stresses [28]. Stress responses are generally associated with a dormancy state in *M. tuberculosis* [29,30]. However, neither *devR* nor *devS* (two genes known to be involved in this phenomenon) were differentially expressed by *M. ulcerans* during the healing stage.

Finally, we investigated transcript levels for genes required for mycolactone biosynthesis. At the healing stage, during which mycolactone was not detected, *M. ulcerans* had unaffected levels of expression (*mup038c*) or a tendency toward higher levels of expression for genes involved in mycolactone synthesis (*mlsA1, mlsA2, mlsB, mup045*), with *mup045* (FC = 1.7, FDR = 0.026) being the only gene significantly upregulated. In light of these results, the decrease in mycolactone production observed during the healing stage was not due to the downregulation of genes encoding proteins involved in mycolactone synthesis.

Our results suggest that *M. ulcerans* has to adapt to a new tissue environment, with different nutrient sources, and that it does so by modifying its metabolic activity, leading to lower levels of mycolactone production.

## Changes in the lipid-associated phenotype of *M. ulcerans* in the mouse skin environment

**Figure 2.**
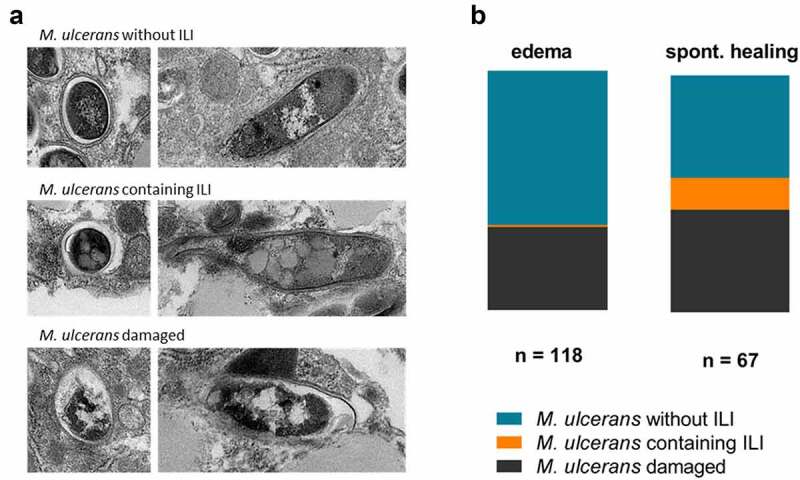
**Changes in the lipid-associated phenotype of *M. ulcerans* during spontaneous healing**. (a) Three bacillus populations were observed by transmission electron microscopy (TEM) in glutaraldehyde-fixed mouse tissues at the edema and healing stages (*n* = 2 mouse/stage): *M. ulcerans* without ILI, *M. ulcerans* containing ILI and damaged *M. ulcerans*. (b) The proportion of *M. ulcerans* bacteria containing ILI increased during spontaneous healing, reaching 13% of total bacillus counts (*n* = 67), versus 0.8% at the edema stage (n = 118)

Previous studies have reported changes in the morphology of mycobacteria and a loss of their acid-fast character [31] under nutrient starvation stress. In this context, we investigated *M. ulcerans* morphology. We analyzed the ultrastructure of *M. ulcerans* in edematous and healed mouse skin, by transmission electron microscopy. Three kinds of *M. ulcerans* populations were observed in tissues: (i) intact bacteria, (ii) intact bacteria containing intracytoplasmic lipid inclusions (ILIs) and, (iii) altered bacteria (Figure 2a). Interestingly, in healed mouse skin, there was a significantly higher proportion of bacteria containing ILIs than at the edema stage (13% of the total *M. ulcerans* population in healed mouse skin, versus 0.8% at the edematous stage) (Figure 2b). The accumulation of ILIs, as already found in several other mycobacteria, is consistent with the changes in lipidomic pathways observed in transcriptomic analyses. This result suggests that a profound change in metabolism occurs in the bacillus during the spontaneous healing of *M. ulcerans* infection, structurally characterized by the accumulation of ILIs.

## Modulation of mycolactone synthesis by carbon source

**Figure 3.**
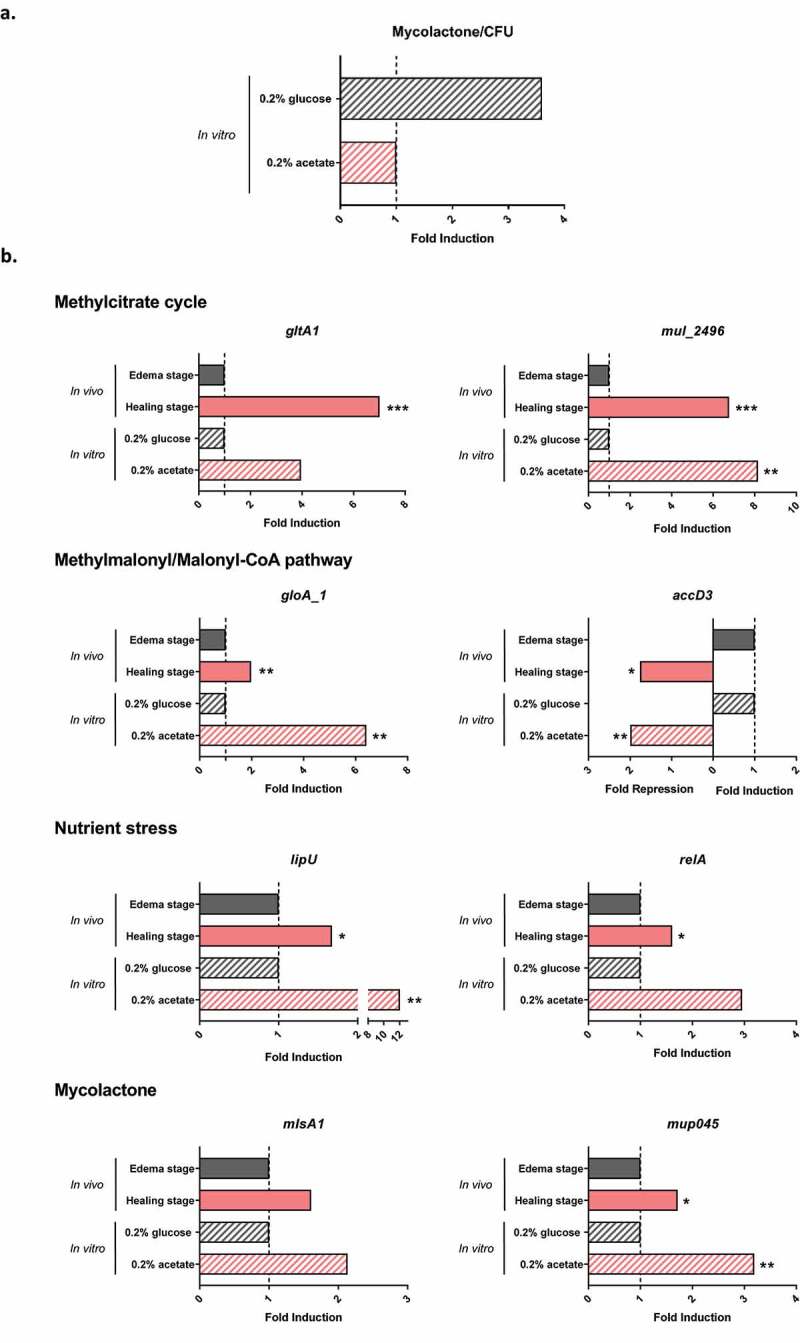
**Regulation of mycolactone synthesis under nutrient variation is associated with an induction of lipid metabolism and stress response pathway genes**. (a) The amount of mycolactone produced by *M. ulcerans in vitro* was 3.7 times higher in the presence of glucose. The results are expressed as mean of two independent experiments. (b) Validation of RNA-seq data with *in vitro* cultures, based on an analysis of gene expression. The expression of genes involved in the methylcitrate cycle, methylmalonyl/malonyl-CoA pathway, nutrient stress and mycolactone synthesis was compared between conditions of high levels of mycolactone production (edema *in vivo* and 0.2% glucose *in vitro*) and low levels of mycolactone production (healing stage *in vivo* and 0.2%acetate *in vitro*). The *in vivo/in vitro* results are expressed as fold induction/repression. For *in vitro* results, fold-changes in gene expression were calculated by comparison with *M. ulcerans* grown in the presence of glucose, for two independent experiments with two to four biological replicates. Statistical analyses comparing cultures in the presence of acetate and glucose culture were performed with nonparametric Mann–Whitney *U* tests. For *in vivo* and *in vitro* fold changes, significant differences are illustrated as *p < 0.05, **p < 0.01, ***p < 0.001

In order to mimic nutrient conditions thought to be encountered *by M. ulcerans* during healing stage *in vivo*, we developed approaches to experimentally modulate the concentration of propionyl-CoA and acetyl-CoA, two key precursors for several cell wall lipids and mycolactone. We further tested *M. ulcerans* growth in the presence of 0.2% propionate (source of propionyl-CoA) or 0.2% acetate (source of acetyl-CoA) and used 0.2% glucose as control. Growth was considerably slower in propionate-supplemented cultures, as reported in previous studies [32,33], but not in acetate-supplemented medium. As the load of viable *M. ulcerans* remains constant during the spontaneous healing stage, we thus focused our analysis on a comparison of acetate and glucose as alternative carbon sources, in an attempt to understand the origin of the regulation of mycolactone synthesis observed *in vivo*. We thus evaluated the impact of these different carbon sources on mycolactone production and transcriptomic changes. We found that *M. ulcerans* could produce mycolactone with both carbon sources. However, *M. ulcerans* produced 3.7 times more mycolactone on glucose-containing medium than in the presence of acetate (Figure 3a). For validation of the RNA-seq data analysis in our *in vitro* conditions, we selected eight key genes differentially expressed during the healing stage: *gltA1* and *mul_2496* (methylcitrate cycle), *gloA_1* and *accD3* (methylmalonyl/malonyl-CoA pathway), *lipU* and *relA* (nutrient stress), and *mlsA1* and *mup045* (mycolactone synthesis). The results obtained *in vitro* confirmed the RNA-seq data analysis obtained *in vivo* (Figure 3b): all these genes were upregulated in the presence of acetate, with the exception of *accD3*, which was downregulated in both the RNA-seq *in vivo* analysis and *in vitro* conditions. These results suggest that *M. ulcerans* adapts its metabolism to its culture conditions, particularly regarding nutrient conditions (absence of glucose), probably reflecting its adaptation in host tissues.

**Figure 4.**
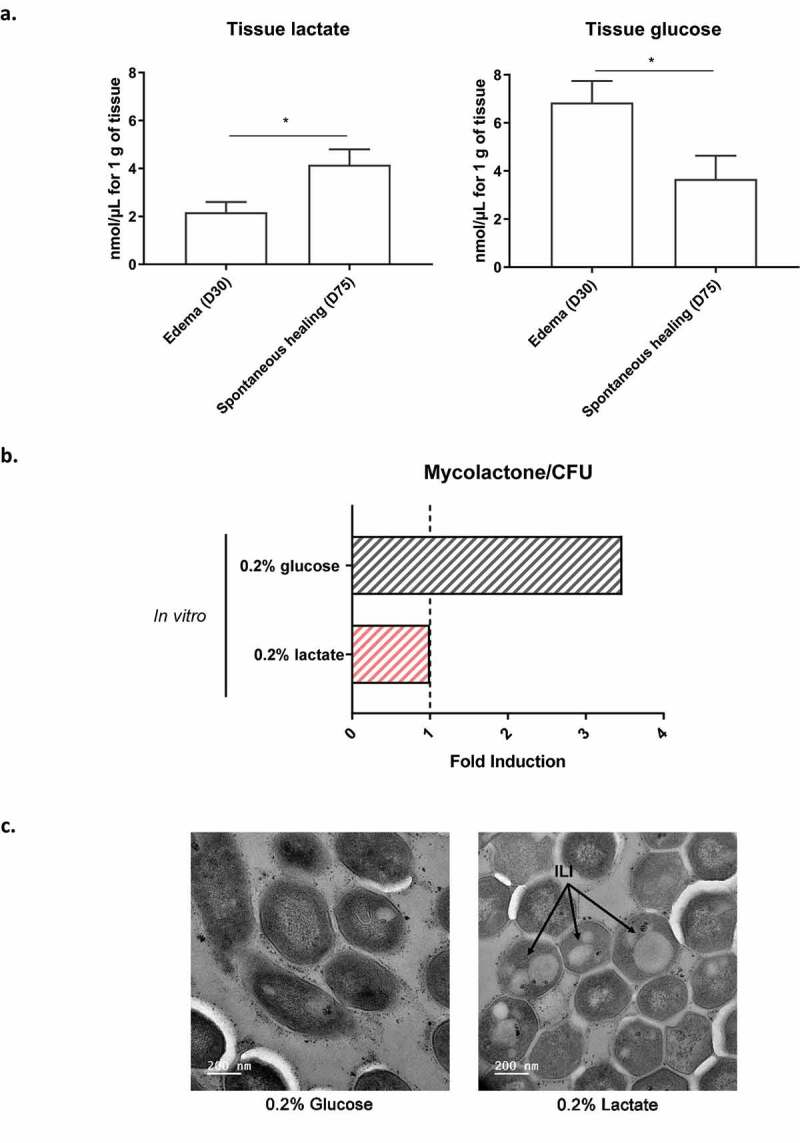
**Lactate availability promotes changes in lipid metabolism of *M. ulcerans***. (a) An increase in lactate availability, and a simultaneous decrease in glucose availability are observed in tissues during spontaneous healing. The amounts of lactate and glucose are expressed per 1 g of tissue (*n* = 8 mice for each stage, *p-value<0.05, Mann–Whitney *U* test). (b) The amount of mycolactone produced by *M. ulcerans in vitro* was 3.5 times higher in the presence of glucose. The results are expressed as mean of two independent experiments. (c) An accumulation of ILIs were observed by transmission electron microscopy (TEM) in the presence of lactate. Black arrows indicate the ILIs in *M. ulcerans* cells

Several studies have shown that wounded tissues have higher rates of glucose utilization and lactate production than intact skin [34–36]. We therefore evaluated the levels of lactate and glucose in healed tissues, comparing the results obtained with those for the edema stage of *M. ulcerans* infection. Lactate concentration was significantly higher (2.2-fold) in healed tissues (4.07 nmol/µL for 1 g of tissue) than in edematous tissues (1.88 nmol/µL for 1 g of tissue) (Figure 4a). By contrast, glucose concentration was significantly lower (2.2-fold) at the healing stage (2.96 nmol/µL for 1 g of tissue) than at the edematous stage (6.64 nmol/µL for 1 g of tissue) (Figure 4a). Thus, lactate availability increases as glucose decreases in tissues, consistent with the hypothesis of a restriction of bacterial access to glucose. In this context, we evaluated the impact of lactate *in vitro*, as potential carbon source, on mycolactone production and *M. ulcerans* morphology. As already observed with acetate, *M. ulcerans* produced 3.5 times more mycolactone on glucose-containing medium than in the presence of lactate (Figure 4b). We next analyzed the ultrastructure of *M. ulcerans* by transmission electron microscopy. In the presence of lactate, *M. ulcerans* was structurally characterized by the accumulation of ILIs (Figure 4c). This result is consistent with the lipid-associated phenotype of *M. ulcerans* observed in healed mouse skin.

Glucose/nutrient availability may, therefore, be a key element in the inhibition of mycolactone synthesis during the healing process. The low mycolactone production profile was associated with (i) an upregulation of genes encoding proteins involved in mycolactone synthesis, (ii) an induction of lipid metabolism pathways (MCC and MMC), (iii) a stress response profile, and finally, (iv) an accumulation of ILIs.

## Discussion

Buruli ulcer is a cutaneous infectious disease caused by *M. ulcerans* and characterized by the development of large ulcerative lesions. These lesions are associated with the ability of this bacterium to produce a lipid toxin, mycolactone, its major virulence factor. *M. ulcerans* infection has three stages in humans: (i) an early pre-ulcerative stage, in which nodules, plaques, or edemas form, (ii) an ulcerative stage, during which lesions progress toward massive ulceration, and (iii) spontaneous healing, which is observed in 5% of Buruli ulcer patients without treatment, and may lead to severe sequelae. We previously showed, in a mouse model reproducing each stage of infection from early lesions to spontaneous healing, that the bacteria remained viable and were present at high loads in healed tissues. Surprisingly, mycolactone was undetectable at this stage. This model provided us an opportunity to study the response of the bacillus and the regulation of mycolactone synthesis.

Dormancy is a well-known bacterial response to environmental change and has been reported to occur in *M. tuberculosis* during latent stage [37–40]. We show here that the dormancy regulon of *M. ulcerans* was not upregulated, even though the transcriptional profile observed resembled a stress response. By contrast, we observed an upregulation of *mprA*, encoding a protein involved in *M. ulcerans* persistence [41], suggesting that the bacterium enters in a phase of persistent infection.

We report here a strong regulation of the fatty-acid metabolism, a key element in mycobacteria, as about 6% of the mycobacterial genome consists of lipid metabolism enzymes [42]. Bacterial nutrition is a key aspect of host-pathogen interaction [43,44], and *M. tuberculosis* is known to use fatty acids as major carbon and energy sources *in vivo*. The catabolism of fatty acids releases acetyl- and propionyl-CoA, to fulfil basic functions: (i) the generation of energy and biosynthetic precursors through central cellular metabolism (TCA cycle), and (ii) lipid biosynthesis through carboxylation by acyl-CoA carboxylases (ACCases). Our results highlighted the induction of the methylcitrate cycle (MCC) known to mediate carbon flux into the TCA cycle, and the methylmalonyl-CoA pathway (MMC), primarily involved in the generation of precursors (methylmalonyl-CoA) for lipid synthesis. Interestingly, we observed an upregulation of *gloA*_*1*, encoding a methylmalonyl-CoA epimerase involved in the epimerization of S-methylmalonyl-CoA into R-methylmalonyl-CoA, a molecule with the wrong stereochemistry for incorporation into polyketides [45]. This result suggests that *M. ulcerans* use the methylmalonyl-CoA pathway for energy production via the TCA cycle, despite the generation of precursors for lipid biosynthesis. In parallel, we found that *accD3* was downregulated. This gene encodes an ACCase involved specifically in the synthesis of malonyl-CoA, a second precursor used for lipid biosynthesis, suggesting that acetyl- and propionyl-CoA enter the TCA cycle directly, to provide the bacteria with sufficient energy. Finally, we observed a strong induction of *mul*_*0949*, encoding a potential sugar transporter, possibly due to nutrient deficiency, a hypothesis supported by the induction of *lipU*, encoding a putative lipase known to be upregulated in conditions of nutrient stress in *M. tuberculosis*.

The adaptation of *M. ulcerans* to its new environment was reflected by a larger proportion of bacteria containing ILIs during healing than at the edema stage. The incorporation of host-derived fatty acids into mycobacterial lipid inclusion [46–49], or directly into mycobacterial membranes [50] has already been reported. Thus, in times of starvation, *M. ulcerans* may hydrolyze stored lipids to satisfy its nutrition and energy requirements, as already suggested for *M. tuberculosis* during latency and reactivation [50–53]. This hypothesis is consistent with the induction of the methylcitrate cycle observed in transcriptomic analyses *in vivo.*

Globally, these results demonstrate the occurrence of profound metabolic changes in the bacillus during spontaneous healing. We hypothesize that the changes observed were linked to the regulation of mycolactone synthesis, because this lipid toxin was not detectable at this stage [16]. Mycolactone is synthesized by type I polyketide synthases (PKS) encoded by a giant plasmid (pMUM001): the *mlsA1, mlsA2* genes for lactone core synthesis and the *mlsB* gene for polyketide chain synthesis. These three PKS contain several consecutive extension modules allowing the incorporation of malonyl- or methylmalonyl-CoA for chain elongation. Two other accessory genes, *mup038* and *mup045*, must also be expressed for mycolactone synthesis. We observed no change in the expression of these two genes, and, indeed, their expression levels were even slightly higher. Thus, mycolactone regulation is not under transcriptional control, as already reported *in vitro* [54]. Several hypotheses may explain the regulation of mycolactone production, including the possible degradation of the toxin, or the limitation of substrates.

In this context, we demonstrated that different carbon sources, such as glucose, acetate, and lactate, affected mycolactone production in *M. ulcerans*. Glucose is the major carbon and energy source for most cells, and is used for glycolysis, the metabolic pathway that converts glucose into pyruvate, which can be further metabolized into acetyl-CoA for the synthesis of polyketides and energy through the TCA cycle. Acetate or lactate may be preferentially metabolized by the TCA cycle for energy synthesis and may simultaneously be directed into the gluconeogenic flux. Gluconeogenesis is essential for the synthesis of amino acids and sugars, such as trehalose, by the pathogen, these compounds being essential for cell wall biosynthesis, and for the synthesis of purines and pyrimidines to build nucleic acids. Thus, in the absence of glucose, *M. ulcerans* had to adapt its metabolism to the culture conditions, by switching to a low mycolactone production phenotype to ensure its replication, as observed during the spontaneous healing process.

We previously showed that spontaneous healing was associated with profound changes, leading to complete tissue remodeling, with an accumulation of myeloid cells, finally resulting in a completely new environment for the bacilli [16]. We show here that glucose levels are lower and lactate levels are higher in healed tissues than in edematous tissues, suggesting high rates of glucose consumption by host cells at this stage, a phenomenon previously shown to be associated with cell proliferation [55–58], operating here during the healing process. Several studies showed that *M. tuberculosis* can co-metabolize multiple carbon sources [59,60], including lactate [61,62]. In our context, lactate could be one possible carbon source for *M. ulcerans* in healed tissues, a hypothesis supported by the induction of the *lldD2* gene (Additional file 2), encoding for a potential quinone-dependent L-lactate dehydrogenase.

In conclusion, this study provides a first description of the transcriptomic profile of *M. ulcerans* during infection in a mouse model. We show that the local microenvironment in healed tissues, which is poor in nutrients, modifies the metabolism of the bacterium, strongly inducing lipid metabolism. In this environment, *M. ulcerans* seems to use host-derived lipids and/or lactate as carbon sources for the production of energy and to fuel biosynthetic pathways. One hypothesis could be a limitation of substrate (malonyl-, methylmalonyl-CoA) for the synthesis of mycolactone. Therefore, *M. ulcerans* has to reduce its secondary metabolism to ensure its survival, and this leads to an inhibition of mycolactone production.

## Data Availability

All relevant data are within the manuscript, its supporting information files and openly available in GEO of NCBI, reference number GSE157956.
